# RETRACTED: Mohamed et al. The Synergistic Hepatoprotective Activity of Rosemary Essential Oil and Curcumin: The Role of the MEK/ERK Pathway. *Molecules* 2022, *27*, 8910

**DOI:** 10.3390/molecules31213795

**Published:** 2026-10-22

**Authors:** Maged E. Mohamed, Nancy S. Younis, Hossam S. El-Beltagi, Omar M. Mohafez

**Affiliations:** 1 Department of Pharmaceutical Sciences, College of Clinical Pharmacy, King Faisal University, Al-Ahsa 31982, Saudi Arabia; nyounis@kfu.edu.sa; 2 Department of Pharmacognosy, Faculty of Pharmacy, University of Zagazig, Zagazig 44519, Egypt; 3 Zagazig University Hospitals, Zagazig 44511, Egypt; 4 Agricultural Biotechnology Department, College of Agriculture and Food Sciences, King Faisal University, Al-Ahsa 31982, Saudi Arabia; 5 Biochemistry Department, Faculty of Agriculture, Cairo University, Giza 12613, Egypt; 6 Department of Biochemistry, Faculty of Pharmacy, University of Al-Azhar, Assiut Branch 71524, Egypt; omarmohafez@azhar.edu.eg

The journal retracts the article titled “The Synergistic Hepatoprotective Activity of Rosemary Essential Oil and Curcumin: The Role of the MEK/ERK Pathway” [1], cited above.

Following publication, concerns were brought to the attention of the Editorial Office regarding the integrity of images presented in this publication [1].

In accordance with standard journal procedures, the Editorial Office and Editorial Board conducted an investigation that identified indications of inappropriate editing and duplication within Figures 3 and 7 presented in this article, as well as between Figure 3 of this article and images of an earlier publication [2], produced by similar authorship groups, reporting different experimental conditions. Although the authors cooperated with the investigation, the explanations and supporting materials provided were not deemed sufficient by the Editorial Board to resolve the identified concerns. As a consequence, the Editorial Board has lost confidence in the reliability of the reported findings and has decided to retract this publication in accordance with MDPI’s retraction policy.

This retraction was approved by the Editor-in-Chief of the journal *Molecules*.

The authors have been informed of this retraction and have indicated their disagreement with this decision.
